# Polyacrylic Acid-Coated LaB_6_ Nanoparticles as Efficient Sensitizers for Binary Proton Therapy

**DOI:** 10.3390/pharmaceutics17040515

**Published:** 2025-04-15

**Authors:** Mariya S. Ryabtseva, Marina V. Filimonova, Alexander S. Filimonov, Olga V. Soldatova, Anna A. Shitova, Vitaly A. Rybachuk, Irina K. Volkova, Kirill A. Nikolaev, Alexander O. Kosachenko, Sergei N. Koryakin, Dmitry S. Petrunya, Polina A. Kotelnikova, Alexander E. Shemyakov, Danil D. Kolmanovich, Anton L. Popov, Gleb V. Tikhonowski, Anton A. Popov, Anna A. Timakova, Andrey V. Kolobov, Sergey M. Deyev, Andrei V. Kabashin, Irina N. Zavestovskaya

**Affiliations:** 1 P. N. Lebedev Physical Institute of the Russian Academy of Sciences, Leninsky Prospect 53, 119991 Moscow, Russia; d.petrunya@lebedev.ru (D.S.P.);; 2 A. Tsyb Medical Radiological Research Center—Branch of the National Medical Research Radiological Center of the Ministry of Health of the Russian Federation, 249036 Obninsk, Russia; mari_fil@mail.ru (M.V.F.); 89208861291@mail.ru (O.V.S.); annaredrose@mail.ru (A.A.S.); rybachukvitaliy@gmail.com (V.A.R.); ik_volkova@mail.ru (I.K.V.);; 3 Obninsk Institute for Nuclear Power Engineering—Branch of the National Research Nuclear University MEPhI, 249040 Obninsk, Russia; 4 Moscow Engineering Physics Institute, National Research Nuclear University MEPhI, Kashirskoe Shosse 31, 115409 Moscow, Russia; 5 Shemyakin-Ovchinnikov Institute of Bioorganic Chemistry, Russian Academy of Sciences, 117997 Moscow, Russiabiomem@mail.ru (S.M.D.); 6 Institute of Theoretical and Experimental Biophysics, Russian Academy of Sciences, 3 Institutskaya St., 142290 Pushchino, Russia; 7 Institute for Regenerative Medicine, Sechenov First Moscow State Medical University (Sechenov University), 8-2 Trubetskaya St., 119991 Moscow, Russia; timakova_a_a@staff.sechenov.ru; 8 LP3, CNRS, Aix-Marseille University, 13288 Marseille, France; 9 National Research Center «Kurchatov Institute», Academician Kurchatov Square 1, 123182 Moscow, Russia

**Keywords:** proton beam therapy, lanthanum hexaboride, nanoparticles, cytotoxicity, tolerability, antitumor efficacy

## Abstract

Proton beam therapy (PBT) is a rapidly advancing modality of hadron therapy. The primary advantage of proton therapy lies in a unique depth-dose distribution characterized by the Bragg peak, which enables a highly targeted irradiation of the area limited to the tumor, while minimizing the impact on healthy tissues. However, a broader clinical adoption of the ion beam therapy is limited by both economic and radiobiological constraints. One of the possible ways to increase the relative biological effectiveness (RBE) of proton therapy involves the use of radiosensitizers. **Background/Objectives**: In this work, we investigated the efficacy of using colloidal solutions of lanthanum hexaboride (LaB_6_) nanoparticles (NPs) coated with polyacrylic acid (PAA) as sensitizers to increase the antitumor biological effectiveness of proton irradiation. This material has not yet been studied extensively so far, despite its promising physical and chemical properties and several reports on its biocompatibility. **Methods**: LaB_6_ NPs were synthesized by femtosecond pulsed laser ablation, functionalized with PAA and characterized. The safety of NPs was evaluated in vitro using a Live/Dead assay on cell cultures: EMT6/P, BT-474, and in vivo in Balb/c mice after intravenous (i.v.) administration. The efficacy of binary proton therapy was evaluated in vitro on cell cultures: EMT6/P, BT-474, and in vivo in the model of human ductal carcinoma of the mammary gland BT-474 in female Nu/j mice after intratumoral (i.t.) administration at a dose of 2.0 mg/mouse and local proton irradiation (fractional exposure of 31 Gy + 15 Gy). The biodistribution of LaB_6_-PAA NPs in the animal body was also evaluated. **Results**: Significant enhancement in cancer cell death following proton beam irradiation was demonstrated in vitro on EMT6/P, BT-474 cell lines. Although the antitumor efficacy observed in vivo was comparatively lower—likely due to the high sensitivity of the BT-474 xenografts—both proton monotherapy and binary treatment were well tolerated. **Conclusions**: LaB_6_-PAA NPs show promise as efficient sensitizers capable of enhancing the biological efficacy of proton therapy, offering a potential path forward for improving therapeutic outcomes.

## 1. Introduction

The urgent need for radiobiological research, which aims to improve tumor control while minimizing the risk of toxic effects on healthy tissues and reducing late complications, has driven the rapid development of cancer treatment using accelerated beams of charged particles (hadron therapy) in recent decades [1]. Proton and ion beam therapy have the potential to become the leading approach for the non-invasive treatment of various cancer types in the early decades of the 21st century. This trend is supported by the nearly exponential growth of both operational and under-construction hadron therapy centers worldwide over the past decade. According to the Particle Therapy Co-Operative Group (PTCOG), as of early 2025, there are 145 hadron therapy centers established worldwide, with 35 additional centers under construction and another 35 in the design phase [2]. Consequently, by early 2028, there will be 215 hadron therapy centers in operation globally. Notably, 196 of these are proton beam therapy centers, which have emerged as the fastest-growing method of hadron therapy, with approximately 50,000 patients treated annually using this method worldwide.

The main reason for the development of research in this area is related to the physical properties of charged particles, which can release energy much more selectively than photons. The depth-dose distribution of protons, described by the Bragg curve [3], enables a significant reduction in the irradiation of healthy tissues along the beam path, while the main part of the dose is sharply concentrated at the end of the particle path—known as the Bragg peak. This allows for the delivery of high-dose gradients close to critical organs, while limiting the high-dose region to the tumor volume. Although there is a lack of randomized clinical trials conclusively demonstrating the advantage of proton therapy over the photon one [4], and there are ongoing debates about cost-effectiveness [5], current results from phase I/II clinical trials support the validity of this approach [6]. This is particularly relevant for deep-seated tumors located near critical organs, as well as for inoperable or recurrent tumors [7]. Proton therapy also remains one of most promising options for pediatric patients due to is ability to significantly reduce the total dose delivered to the body [8], even in comparison with new photon-based techniques such as intensity modulated radiotherapy (IMRT) [9].

Despite the ongoing advancements in proton beam therapy and the previously mentioned advantages, standard clinical practice continues to assume a relative biological effectiveness (RBE) of protons at approximately 1.1. This value is significantly lower than that of heavy ions, such as carbon ions (^12^C), which exhibit RBE values ranging from 1.5 to 5 [10]. However, the high cost of installations required for carbon ion therapy currently limits its widespread clinical use. Consequently, the enhancement of the therapeutic efficacy of proton beam therapy necessitates the development of technologies that increase the likelihood of cancer cell death during irradiation [11,12].

One promising avenue involves the use of radiosensitizers, which are chemical or pharmacological agents that enhance the damaging effect of ionizing radiation on cancer cells [13]. Over recent decades, numerous studies have explored the radiosensitizing and synergistic effects of nanoparticles (NPs) in radiotherapy [14]. Over recent decades, numerous studies have explored the radiosensitization and synergistic effects of nanoparticles (NPs) in radiotherapy [14]. A widely used strategy entails enhancing therapeutic outcomes by incorporating high atomic number (Z) metal NPs, such as gold (Au-NPs) [15,16,17], iron (Fe-NPs) [18], platinum (Pt-NPs) [19], and bismuth (Bi-NPs) [20,21]. The first studies investigating the combination of NPs with protons date back to 2010, when Kim J.-K. et al. demonstrated, through both in vitro and in vivo experiments, the enhanced anti-tumor efficacy of proton irradiation achieved by introducing small Fe and Au-NPs (2–15 nm) [22]. Follow-up studies by the same group in 2012 [23] further highlighted the substantial therapeutic potential of metal NPs in proton therapy, illustrating the multicomponent nature of their combined effects with proton radiation. Our previous research has demonstrated the effectiveness of Bi [20,21] and Au-NPs [24], both in vitro and in vivo settings. In one study [20], Bi-NPs induced considerable apoptotic cell death after proton irradiation at the Bragg peak. The maximal therapeutic effect was observed at a radiation dose of 3 Gy and a NP concentration of 50 μg/mL, resulting in a 97% reduction in tumor cell clonogenic activity. These findings were validated in vivo using a model of aggressive Sa37 sarcoma in mice, where a 60% inhibition of primary tumor growth was recorded, along with a decreased metastatic activity in popliteal and axillary lymph nodes. In another study [24], a dose of 4 Gy combined with Au-NPs at concentrations above 25 mg/l resulted in the complete eradication of EMT6/P cells in vitro. Furthermore, binary proton therapy employing targeted Au-NPs led to an 80% inhibition of tumor growth in vivo. The mechanism underlying the enhancement of proton irradiation by metal NPs is typically attributed to the generation of reactive oxygen species (ROS) induced by proton irradiation. ROS are formed through water radiolysis surrounding the NPs, the emission of secondary electrons from the NPs [25], or catalytic processes at the NP/water interface [26]. ROS are well known for their ability to effectively destroy cancer cells [27], thereby significantly enhancing the efficacy of proton therapy [19,28].

An alternative strategy for enhancing proton therapy is the use of boron-based sensitizers. Several studies have demonstrated that boron-containing compounds can substantially improve the therapeutic efficacy of proton irradiation [29,30,31,32]. For example, Cirrone et al. [29] used sodium borocaptate (BSH) at concentrations of 40 and 80 ppm to enhance the sensitivity of DU145 prostate cancer cells to proton irradiation. Upon irradiation with protons at 62 MeV, a pronounced enhancement of the cytogenetic effects was observed. FISH analysis revealed a significant increase in the number of chromosomal abnormalities in the presence of BSH. In a separate study involving an in vivo glioblastoma model [30], researchers confirmed the benefits of incorporating boron into proton therapy. Employing combined micro-PET/CT imaging in real time, it was demonstrated that boron usage significantly enhances the therapeutic outcome compared with conventional techniques, facilitating increased tumor cell death and promoting mitophagy processes. In our study [31], we evaluated the efficacy of boron NPs (B-NPs) in killing cancer cells during 160.5 MeV proton beam irradiation. Irradiating MNNG/Hos cells at a dose of 3 Gy in the presence of B-NPs at concentrations of 80 and 100 ppm reduced cell colony formation by factors of 2 and 2.7, respectively, compared with control samples irradiated without B-NPs.

Despite the observed effects of increased cancer cell lethality, the precise mechanisms of boron-enhanced proton therapy remain a topic of ongoing debate. Some researchers believe in a nuclear mechanism, which implies the formation of high LET α-particles through the nuclear reaction p + ^11^B → 3α [29,30,32]. This process could potentially lead to irreversible double-strand DNA damage in cancer cells, similar to boron neutron capture therapy (BNCT) [33]. However, others question the viability of this reaction and suggest that similar biological outcomes could be achieved using X-rays, implying that nuclear reactions may not play a significant role [34]. Additionally, several studies using Monte Carlo simulations convincingly demonstrated that the contribution of generated α-particles to the overall radiation dose is relatively low [35,36]. Sensitivity appears to vary based on cell type, cultivation conditions, boron metabolism, or the bystander effect [34]. Alternative mechanisms involving secondary thermal neutrons generated during the proton beam interaction with the target have also been proposed [36,37], but these mechanisms are also a subject of debate [32]. In our previous study [38], we showed that radiosensitization might result not only from nuclear reactions but also from increased ROS production, alterations in the cellular redox status, and enhanced oxidative stress, ultimately leading to apoptosis. It appears that the radiosensitizing effect of boron-containing compounds in boron–proton interactions is based on a complex mechanism that includes both the generation of alpha particles and redox processes, which initiate the development of oxidative stress, leading to the apoptosis of cancer cells.

Given the demonstrated efficacy of both high-Z metal nanoparticles and boron-containing compounds in enhancing proton therapy, a logical step is to explore materials that combine the advantages of both approaches. In this work, we have, for the first time, experimentally assessed the potential of lanthanum hexaboride (LaB_6_) NPs, functionalized with polyacrylic acid (PAA), to enhance the biological efficacy of protons specifically within the tumor region. Lanthanum hexaboride NPs are not yet widely utilized, although some publications demonstrate their high biocompatibility [39]. Their potential advantages include a low work function (2.50 eV), degradability in aqueous environment, small size after the synthesis, a relatively high atomic number of lanthanum (Z = 57), and finally, unique optical properties [40]. These properties open up attractive opportunities for enabling both therapy (proton, photon-capture, photothermal, and gamma therapy) and diagnostic (computer tomography and photoacoustic visualization) modalities.

## 2. Materials and Methods

### 2.1. Synthesis and Functionalization of NPs

Polyacrylic acid (PAA)-functionalized lanthanum hexaboride NPs (LaB_6_-PAA NPs) were used in this work. Colloidal solutions of spherical LaB_6_ NPs were obtained by femtosecond pulsed laser ablation of a solid lanthanum hexaboride target in deionized water. The surface of LaB_6_ NPs was functionalized with PAA. For this purpose, 2.1 kDa polyacrylic acid (Sigma, Tokyo, Japan) (250 mM) was dissolved in 10 mL of mQ water and heated up to 60 °C for 15 min, after which the polymer was added to 100 mL of NP solution at a concentration of 0.15 g/L in mQ water, thoroughly mixed and incubated for 20 min at 25 °C. The colloidal solution was then concentrated, washed to remove low molecular weight compounds, and buffer-exchanged using a JetBio-Filtration centrifugal concentrator (50 kDa) at 4000 g.

### 2.2. Characterization of LaB_6_-NPs

The concentration of LaB_6_-PAA NPs was estimated by ICP-MS for lanthanum after dissolution in nitric acid using a NexION 2000 mass spectrometer (PerkinElmer, Shelton, CT, USA). The optical extinction spectrum was determined by optical spectroscopy using an Infinite M1000 PRO analyzer (Tecan, Salzburg, Austria). The morphology and size distribution of NPs were determined by a SEM (MAIA 3, Tescan, Brno–Kohoutovice, Czech Republic) and TEM system (JEM 2010, JEOL, Peabody, MA, USA) operating at 200 kV. The hydrodynamic size was determined by dynamic light scattering (DLS) using a Zetasizer Nano ZS analyzer (Malvern Instruments Ltd., Malvern, UK). The measurement was performed in deionized water, PBS, and DMEM medium (PanEco, Moscow, Russia) with 10% FBS (HyClone, Cytiva, Pasching, Austria). The ζ-potential of the NPs was measured in an aqueous solution of NaCl (10 mM) at 25 degrees by electrophoretic light scattering (using the Smoluchowski approximation) using a Zetasizer Nano ZS analyzer (Malvern Instruments Ltd., Malvern, UK). NP degradation was evaluated by the release of lanthanum ions under various conditions. For this purpose, NP solutions were prepared at a concentration of 0.1 g/L in distilled water, PBS (100 mM, pH 7.4, composition: 90 mM of NaCl, 1.8 mM of KCl, 7 mM of Na_2_HPO_4_, and 1.2 mM of KH_2_PO_4_), and the simulated lysosomal fluid (SLF, pH 4.5) [20,41], composition: 108 mM of citric acid, 0.5 mM of MgCl_2_, 55 mM of NaCl, 0.5 mM of Na_2_HPO_4_, 0.27 mM of Na_2_SO_4_, 150 mM of NaOH, 0.9 mM of CaCl_2_, 0.3 mM of trisodium citrate, 0.8 mM of glycine, 4.6 mM of sodium tartrate, 0.8 mM of sodium lactate, and 0.8 mM of sodium pyruvate). Then, 300 μL aliquots of samples were taken at 0 and 30 min and 1, 3, 6, and 24 h after solution preparation. The aliquots were centrifuged for 15 min at 20,000× *g* to separate the NPs, and the precipitate and supernatant were analyzed separately for lanthanum content by ICP-MS as described above.

### 2.3. In Vitro Studies

#### 2.3.1. Cell Cultures

For in vitro studies, the following cell cultures were used: mouse carcinoma EMT6/P line (from the European Collection of Authenticated Cell Cultures (ECACC)) and human breast carcinoma BT-474 line (ECACC). The cell lines were cultured in the DMEM/F12 culture medium (PanEco, Moscow, Russia) with the addition of 10% fetal bovine serum (HyClone, Logan, UT, USA), 2 mM of L-glutamine (PanEco, Moscow, Russia), 100 U/mL of penicillin, and 100 µg/mL of streptomycin (PanEco, Moscow, Russia) at +37 °C, 5% CO_2,_ and 95% humidity.

For in vivo studies, a xenograft of the human breast ductal carcinoma BT-474 (obtained from the cell collection of the M.M. Shemyakin and Yu.A. Ovchinnikov Institute of Bioorganic Chemistry, Russian Academy of Sciences, Moscow, Russia) was used. The cell line was cultured in a CO_2_ incubator at 37 °C and 5% CO_2_ in a medium containing 450 mL RPMI (PanEco, Russian Federation), 5 mL streptomycin and penicillin (PanEco, Russian Federation), 300 mg L-glutamine (PanEco, Russian Federation), and 10% bovine serum (PanEco, Russian Federation).

#### 2.3.2. Cytotoxicity

The cytotoxicity of LaB_6_-PAA NPs in vitro was evaluated by analyzing the proportion of live and dead using Live/Dead assay after coincubation with a colloidal solution of NPs on cell cultures: EMT6/P, BT-474, for 16 h (overnight). We used the mixture of the fluorescent dyes Hoechst 33342 and propidium iodide (PI), dissolved in a Hanks’ Balanced Salt Solution (HBSS) (PanEco, Moscow, Russia) at a concentration of 1 µM. After 15 min of incubation with dyes, the cells were washed twice with HBSS, and then the microphotography of the cells was carried out using a ZOE fluorescent cell imager (Bio-Rad, Hercules, CA, USA). The total number of cells and number of dead cells were counted using the ImageJ 1.53 software. After that, the percentage of dead cells was calculated.

#### 2.3.3. Efficacy of Proton Therapy In Vitro

The efficacy of LaB_6_-PAA NPs as a sensitizer to enhance the biological effectiveness of proton irradiation in vitro was evaluated using EMT6/P and BT-474 cell cultures. For each study, cells were seeded in a 12.5 cm^2^ culture flask (SPL, Pocheon, Republic of Korea) as a monolayer on the inner wall measuring 35 × 45 mm and allowed to fully attach for 24 h. The medium was then changed to fresh medium containing LaB_6_-PAA NPs at the required concentration (0–50 µg/mL) or fresh medium without NPs as a control. Cells were incubated with LaB_6_-PAA NPs or without NPs for 16 h before irradiation. Prior to exposure, the cells were washed three times with Hanks’ buffer to remove NPs that did not penetrate the cell and irradiated in a serum-free culture medium.

Irradiation of cell cultures. The irradiation was carried out at the proton therapy complex “Prometheus” (Physico-technical Center of P.N. Lebedev Physical Institute RAS). A pencil scanning proton beam with a fixed energy of 160.5 MeV was directed at the cell vials for irradiation. To precisely position the Bragg peak on the cell monolayer, a 151.8 mm thick polymethyl methacrylate (PMMA) range shifter was installed on the beam path. A uniform field 45 × 55 mm at 95% isodose level was formed to completely cover the cell monolayer. Dose uniformity and field size were controlled using an EBT3 radiometric film, and the absorbed dose was measured using a PTW Unidos electrometer with a PTW microDiamond 60019 ionization chamber. The absorbed dose was monitored before the irradiation of the culture flasks both in front and behind the PMMA range shifter. The flask was completely filled with the culture medium, and the lid was fixed with parafilm. The vials were positioned vertically at a distance of 80 cm from the accelerator nozzle. The vials were positioned in the irradiation zone using a laser positioner, which indicated the center of the irradiated volume. At the accelerator platform, the cell cultures were stored in a thermostat at 37 °C and were kept at room temperature for no more than 5 min during the time of vial fixation and irradiation.

Irradiation was carried out at doses of 0 Gy, 2 Gy, and 4 Gy sequentially; one by one, the groups followed from the low dose to the higher dose. When moving from one irradiated group to another, the dosimetric control was repeated with the use of ionization chambers. At a isodose level of 95%, the homogeneity was at least 98%. In the Bragg peak, the error in determining the absorbed dose did not exceed 10% due to high dose gradients. The efficacy was estimated using a panel of complementary methods: analysis of apoptotic cells in the culture after coincubation with NPs, analysis of their clonogenic activity, analysis of the mitochondrial membrane potential (MMP), and intracellular ROS level.

Clonogenic assay. The clonogenic activity of cells was evaluated 8 days after irradiation and single-cell seeding. Four hours after irradiation, the irradiated cell monolayer was washed three times with Hanks’ solution and then removed from the culture flask using a 0.25% trypsin/EDTA solution. After counting, the cells were seeded in 6-well plates (well area of 10 cm^2^) at a density of 1000 cells per well and cultured for 8 days. Then the cells were washed three times with Hanks’ solution and fixed with 4% paraformaldehyde solution prepared in PBS. Then, cells were washed three times in PBS to remove residual paraformaldehyde, and were stained with 0.1% crystal violet solution for 15 min. Wells were washed with distilled water for 2–3 min, and the stained colonies were counted. A colony was considered to be a group consisting of more than 50 cells. The coefficient of drug interaction (CDI) was calculated as reported by Bontempo et al. [42] with the following equation:

CDI = (NPs + Irradiation) × nt/(NPs × Irradiation),(1)

where (NPs + Irradiation) is the number of colonies with combined action, nt is the non-treated control group, and «NPs» and «Irradiation» represent the number of colonies in groups treated with each alone. Values ≤ 0.7 are considered synergic interaction; values in the range 0.7–1.3 indicate additive interaction, and values ≥ 1.3 indicate an inhibitory effect.

Analysis of apoptotic cells. To evaluate the level of apoptosis after irradiation, the cells were seeded in 8-well slide flasks (SPL, Pocheon, Republic of Korea) at a density of 30 thousand/cm^2^. The number of apoptotic cells was determined by fluorescence microscopy of slide flasks stained with the selective dye Yo-Pro-1 (Invitrogen, Carlsbad, CA, USA) 72 h after the irradiation. Cells attached to the slide flask were stained in accordance with the manufacturer’s protocol and visualized 25 min after using a Zoe fluorescence inverted microscope (Bio-Rad, Hercules, CA, USA). For each group of cells, at least three fields in each well of the slide flask were examined.

Mitochondrial membrane potential. To evaluate the level of mitochondrial membrane potential, we used a potential-sensitive cationic fluorescent dye, tetramethylrhodamine (TMRE), which easily penetrates the cell membrane and stains the mitochondrial membrane in a potential-dependent manner. Cells were incubated with a medium containing a TMRE solution at a concentration of 1 μM for 20 min. Microphotographs of TMRE stained cells were obtained using a Zoe fluorescent inverted microscope (Bio-Rad, Hercules, CA, USA). For each group of cells, at least three fields in each well of the slide vial were examined.

Intracellular ROS level. Intracellular levels of reactive oxygen species (ROS) were evaluated using the CellROX Green dye (Invitrogen, Carlsbad, CA, USA). The cells were stained 24 h after irradiation by adding 5 μM of the dye. Twenty minutes after staining and subsequent washing of the cells, fluorescence was measured using a BioTek Synergy H1 plate reader (BioTek, Winooski, VT, USA) using an excitation and emission pair of 485/520 nm.

### 2.4. In Vivo Studies

#### 2.4.1. Animals

Female athymic mice Nu/j (2.5–3 months old, body weight of 19–27 g) were used to evaluate the antitumor activity. The animals were purchased from the Russian National Center for Genetic Resources of Laboratory Animals based on the SPF vivarium of the Institute of Cytology and Genetics SB RAS (Novosibirsk), and were placed for acclimatization (during 14 days) and investigation in an SPF box of the A.F. Tsyb Medical Research Center. Mice were kept in T-3 cages with 5–10 individuals under conditions of natural light at a temperature of 27–30 °C and a relative humidity of 40–70%, on Rehofix MK 2000 bedding (JRS, Rosenberg, Germany). The animals had free access to PK-120-1 briquetted feed (Laboratorsnab, Russian Federation) and water. The bedding, briquetted feed, and water were pre-sterilized by autoclaving.

Male Balb/c mice (body weight 27–31 g) were used in the in vivo safety study. The animals were purchased from the Andreevka branch of the Federal State Budgetary Institution of Science “Scientific Center for Biomedical Technologies” of the Federal Medical and Biological Agency of Russia (Moscow, Russia), and were acclimatized during 14 days. The animals were kept in T-3 cages under natural light under standard laboratory conditions (forced ventilation of 16 volumes/hour, temperature of 18–20 °C, relative humidity of 50–70%). The animals had free access to water and the rodent feed PK-120-1 (OOO Laboratorsnab, Moscow, Russia).

The animal studies were approved by the Ethics Committee (protocol 1-H-00064, 2 December 2024) and were conducted in accordance with generally accepted standards for the treatment of animals, European Convention ETS/STE No. 123 and the international GLP standard (OECD Guideline 1:1998).

#### 2.4.2. Safety

The in vivo safety of LaB_6_-PAA NPs was evaluated at doses 0.1 and 2.0 mg/mouse (*n* = 5) intravenously (i.v.), into the lateral tail vein, in compliance with the principles of asepsis and antisepsis. The injection volume was 0.2 mL/mouse. The injection rate was 0.1 mL/15 s. PBS was used as a solvent.

#### 2.4.3. Antitumor Efficacy

The antitumor and radiosensitizing effect of proton radiation in combination with LaB_6_-PAA NPs was evaluated using the xenograft model of human ductal carcinoma of the mammary gland BT-474 with a high expression of HER2 receptors in female Nu/j mice (*n* = 35) by intratumor (i.t.) injection. The animals were randomized by body weight. Suspension of BT-474 carcinoma cells was administrated, inoculated subcutaneously, in the lateral surface of the right thigh at a dose of 4 million tumor cells (day 0). The cell suspension for transplantation was prepared on the basis of RPMI medium with the addition of 40% Matrigengel (300 μL Corning, Glendale, AZ, USA, + 500 μL ABW, Shanghai, China). Three groups of animals were formed: a tumor growth control, a group of animals that received proton therapy without a sensitizing agent, and a group of animals that received binary proton therapy (NPs + protons). The proton therapy complex (PTC) “Prometheus” located in the A.F. Tsyb Medical Research Center was used as the source of the proton beam. For the proton irradiation of the BT-474 xenograft, each tumor-bearing mouse was fixed on the radiobiological stand so that its hind limb with the tumor was lateralized, while the proton beam axis was aligned with the center of the xenograft [43]. The tumors were exposed to the modified proton beam with an energy of 95–105 MeV with a diameter of 12 mm through the polymethyl methacrylate filter, which ensured a uniform irradiation of the target in a modified Bragg curve (peak width of 35 mm). Based on the ICRU 90 corrected values, the estimated range of LET for this SOBP extends from 7.02 to 14.00 MeV·cm^2^/g. The proton therapy course was started on the 19th day after neoplasia transplantation, when the volume of tumor nodes in all mice included in the experiment reached 400–490 mm^3^. A local proton irradiation of BT-474 carcinoma in mice of the experimental groups was performed fractionally: on the 19th day of tumor growth at a dose of 31 Gy and on the 33rd day of tumor growth at a dose of 15 Gy. I.t. injection of LaB_6_-PAA NPs at a dose of 2.0 mg/mouse (0.1 mL) or a buffer solution as a control was made 15 min before the first irradiation. Therapy effectiveness was evaluated during 26 days after irradiation. The survival of mice and the dynamics of BT-474 carcinoma xenograft development in mice were evaluated. The morphometry of the carcinoma xenograft was evaluated starting from the 19th day after BT-474 transplantation and then every 2–3 days till the end of the observation period in all tumor-bearing mice used in the study. The linear orthogonal dimensions of the tumor node were measured using the digital caliper ChZ-1-125 (ChIZ, RF) with an accuracy of 0.1 mm. The volumes of tumor nodes were assessed using the method previously described in [44].

For antitumor effect analysis, relative volumes were calculated and normalized to the tumor volume in a given animal on the day of the start of experimental therapy. On the 26th day after irradiation (45th day after tumor transplantation), the animals were euthanized using a CO_2_ euthanizer (AWTech, Moscow, Russia). Necropsy was performed, internal organs (liver, lungs, spleen, kidneys, heart) and tumor nodes of BT-747 carcinoma were isolated and weighed using an Explorer Ex124 analytical scale (Ohaus Corp., Parsippany, NJ, USA). The part of the tissues was fixed in 10% neutral buffered formalin (BioVitrum, Russia) for further histological examination. The remaining tissues were frozen at −20 °C in individual tubes and used to evaluate the systemic availability of LaB_6_-PAA NPs from the tumor node.

#### 2.4.4. Biodistribution

The systemic availability and biodistribution of LaB_6_-PAA NPs from the tumor node were evaluated by the accumulation of lanthanum in tissues. For this purpose, a 5-fold volume of concentrated nitric acid was added to each organ. Samples were heated at 80 °C for 1 h and at 25 °C for 24 h. After the samples were dissolved in deionized water to 20% acid content. The content of lanthanum ions was measured in dissolved organs by ICP-MS using a NexION 2000 mass spectrometer (PerkinElmer, Springfield, IL, USA). The concentration of lanthanum in individual organs or the total mass of lanthanum per organ was calculated.

#### 2.4.5. Morphology

Morphological studies of tissues were performed after fixation in 10% formalin solution using the standard histological technique, a section thickness of 6 μm, hematoxylin and eosin staining. The histological specimens were examined at the Institute of Regenerative Medicine of the First Moscow State Medical University, named after Sechenov (Sechenov University). A LEICA DM4000 B LED microscope equipped with a LEICA DFC7000 T digital camera running LAS V4.8 software (Leica Microsystems, Wetzlar, Germany) was used to examine and visualize the samples. Histological features of antitumor efficacy were described in each tumor node. Necrosis, tumor parenchyma area, and microenvironmental cells were counted. The mitotic index was calculated by examining 10 fields of view in each section at a magnification of ×400, and the number of mitotic figures per mm^2^ was determined.

### 2.5. Statistical Analysis

The results of in vitro experiments were processed in Microsoft Excel 2017 and GraphPad Prism 8.0. Data are presented as mean ± SD. Reliability was calculated using the Mann–Whitney U test and *t*-test.

Statistical analysis of in vivo data was performed using multiple intergroup comparisons by the Kruskal–Wallis ANOVA test with post hoc analysis using the Mann–Whitney U test with Holm–Bonferroni corrections. Statistical calculations were performed using the Statistica 10.0 software package (StatSoft Inc., Tulsa, OK, USA).

## 3. Results

### 3.1. Synthesis, Functionalization, and Characterization of NPs

**Figure 1:**
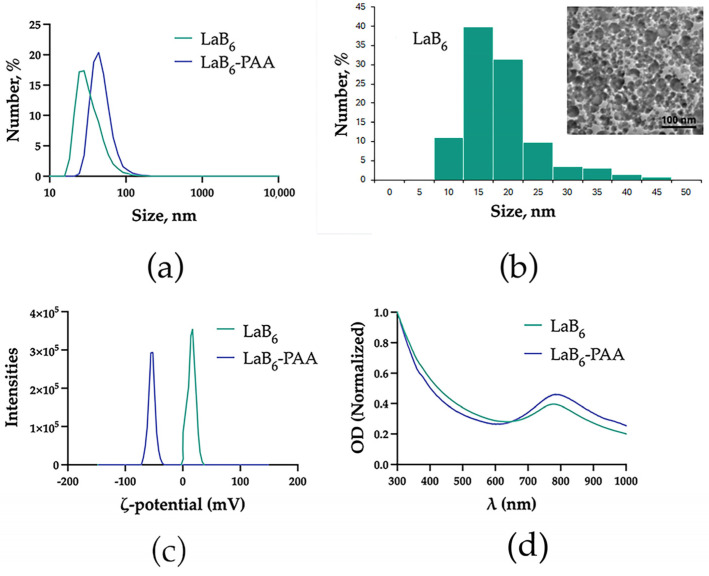
Physical–chemical characterization of NPs: (**a**) hydrodynamic diameter, (**b**) TEM images and size distribution, (**c**) zeta potential, and (**d**) extinction spectra.

Colloidal solutions of the primary LaB_6_ NPs were obtained by femtosecond pulsed laser ablation of the solid lanthanum hexaboride target in deionized water. The primary LaB_6_ NPs had a hydrodynamic size of 35.5 ± 16.3 nm in water, typical optical absorption with the pronounced peak in the middle of the relative transparency window of biological tissue—779 nm (Figure 1) and spherical morphology.

**Figure 2:**
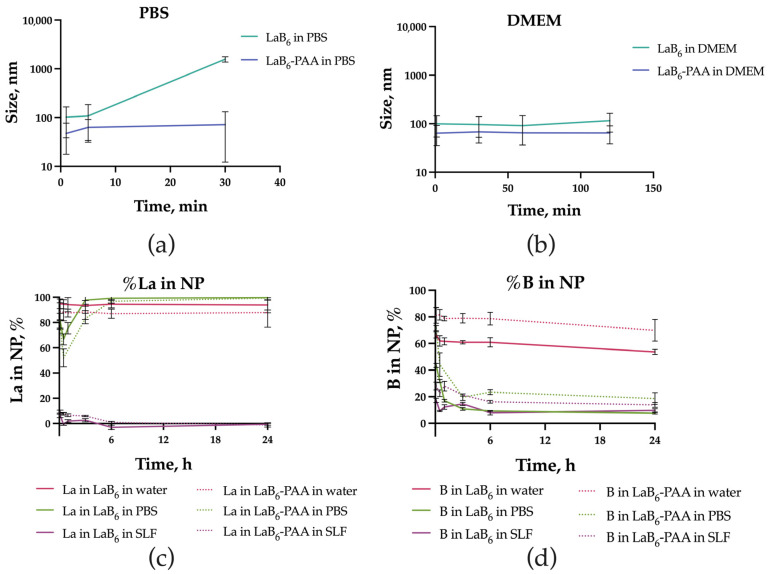
LaB_6_ and LaB_6_-PAA NPs stability in colloidal solutions, including the change in the NPs hydrodynamic diameter over time in (**a**) PBS and (**b**) DMEM +10% FBS and the change in (**c**) lanthanum and (**d**) boron ion presence in NPs in different media over time.

LaB_6_ NP solutions demonstrated colloidal stability in deionized water, which is due to the uncompensated surface charge of laser-synthesized NPs (zeta potential of 16.9 ± 6.3 mV), and chemical stability (Figure 2). However, under physiologically relevant conditions (in PBS), LaB_6_ NPs quickly aggregated: in the 1st minute, they reached a size of 101.8 ± 63.5 nm, after 5 min, 107.8 ± 76.4 nm, and then formed aggregates larger than a micron (Figure 2a). In the DMEM culture medium with 10% FBS, the uncoated nanoparticles showed higher stability, but an increase in size up to 99.5 ± 46.6 nm was observed after a minute of incubation, and a further smooth increase in size up to 96.1 ± 43.7 nm in 30 min, 91.6 ± 54.9 nm in 60 min, and 115.5 ± 48.6 nm in 120 min. After coating with polyacrylic acid (PAA), the hydrodynamic size of the NPs was 45.7 ± 17.4 nm. The extinction spectrum after coating changed insignificantly. The plasmon resonance peak shifted by 5 nm toward the IR region from 779 to 784 nm. The spherical morphology of the particles was preserved. The zeta potential of the NPs in an aqueous solution of NaCl (10 mM) changed from 16.9 ± 6.3 mV to −55.8 ± 5.9 mV, which determined the greater colloidal stability of the NPs under physiologically relevant conditions. In the PBS, the particle size of PAA-coated nanoparticles remained virtually unchanged after 1 min (47.1 ± 29.1 nm), gradually increasing after 5 (42.8 ± 28.8 nm) and 30 min (71.9 ± 59.7 nm). In the DMEM culture medium with 10% FBS, the coated nanoparticles showed even greater stability. The size of LaB_6_-PAA NPs after 1 min of incubation in PBS increased to 63.7 ± 28.2 nm and then did not change significantly, counting 67.9 ± 27.7 nm after 30 min, 64.7 ± 28.3 nm after 60 min, and 64.4 ± 25.9 nm after 120 min. The additional increase in the NPs’ hydrodynamic size under conditions of culture medium may be associated with the formation of a protein corona of serum proteins on the surface of charged NPs.

In distilled water, the degree of NP degradation was insignificant. Most of the lanthanum and boron remained in NPs, changing slightly over time. The transfer of the particles under physiologically relevant conditions accompanied the increase in the rate and completeness of their degradation. Nanoparticles placed in PBS were being dissolved during few hours. Boron ions passed into soluble compounds, remaining in the solution, while lanthanum, over time, precipitated again as part of insoluble reaction products with buffer components. In the acidic SLF medium, the dissolution of NPs was faster and more complete: more than 80% of boron and more than 90% of lanthanum passed into solution. At the same time, both lanthanum and boron remained in the solution as dissolved compounds. Coating the surface of the particles with PAA reduced the rate of their chemical degradation in solutions, but did not prevent it completely.

### 3.2. Cytotoxicity

**Figure 3:**
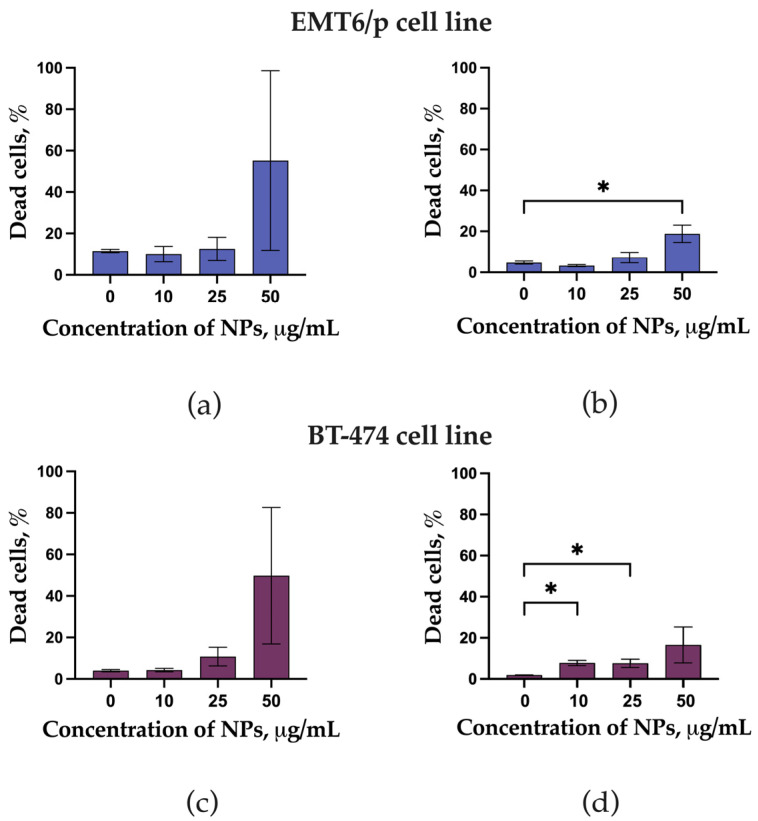
Live Dead assay of EMT6P and BT 474 cell lines after coincubation with LaB_6_-PAA NPs (10–50 µg/mL): (**a**) EMT6P cell lines after 24 h coincubation, (**b**) EMT6P cell lines after 72 h coincubation, (**c**) BT 474 cell lines after 24 h coincubation, and (**d**) BT 474 cell lines after 72 h coincubation. The statistical significance of deviations between the control and test samples is indicated by *, where * *p* < 0.05.

Preincubation of EMT6/P and BT-474 cells with LaB_6_-PAA NPs resulted in a concentration-dependent increase in the number of non-viable cells, which demonstrates the cytotoxic effect of the LaB_6_-PAA nanoparticle for those cell lines even in the absence of irradiation (Figure 3). The cytotoxic effect reveals itself in the detachment of a large part of the cells from the substrate, and this may be associated with a partial dissolution of the nanoparticles and the release of lanthanum ions, which, in turn, may not only have a pronounced cytotoxic effect themselves, but are also capable of replacing calcium ions in the structure of intercellular contacts based on calcium-dependent proteins of the cadherin family, and this leads to their detachment and death. It is worth noting that, after 24 h of coincubation, we observed a higher percentage of dead cells relative to the control as compared with 72 h of coincubation. This phenomenon can be explained by the detachment and death of a large part of cells, followed by their washing at the staining stage.

BT 474 cells appeared to be less sensitive to the cytotoxic effects of LaB_6_-PAA nanoparticles, while EMT6P mouse carcinoma cells were more sensitive to the nanoparticles.

### 3.3. Efficacy of Proton Therapy In Vitro

**Figure 4:**
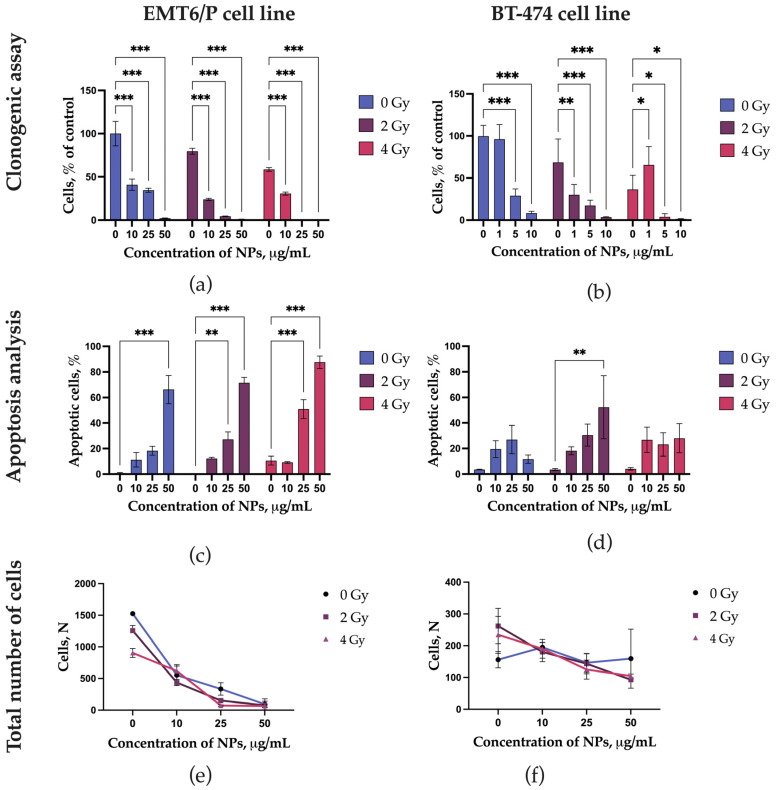
Comprehensive analysis of the radiosensitizing effect of LaB_6_–PAA nanoparticles on EMT6/P and BT-474 cell line cultures, including (**a**,**b**) analysis of clonogenic activity, (**c**,**d**) the proportion of apoptotic cells, and (**e**,**f**) the total number of cells after irradiation, 72 h after irradiation with a proton beam at the Bragg peak. The statistical significance of deviations between the control and test samples, where * *p* < 0.05, ** *p* < 0.01, and *** *p* < 0.001.

LaB_6_-PAA nanoparticles at concentrations of 25 and 50 μg/mL significantly reduces clonogenic activity for both types of cells (Figure 4). At the same time, the EMT6P line showed a significant decrease in clonogenic activity even at a minimum concentration of 10 μg/mL. A similar situation was observed for the part of apoptotic cells after the irradiation in the presence of LaB_6_-PAA nanoparticles. The diagram shows that irradiation at doses of 2 and 4 Gy at the maximum concentration of nanoparticles (50 μg/mL) leads to a significant increase for the part of cells in apoptosis (up to 80%). The BT-474 cell line demonstrated a less pronounced increase of the part of apoptotic cells (about 30–40%). We also noted that some of the cells looked like a “ball” and detached from the substrate after incubation with LaB_6_-PAA nanoparticles and irradiation. Therefore, we additionally analyzed the total number of cells in the culture after the irradiation. The combined effect of proton irradiation and preincubation of the human breast duct cell line BT 474 with LaB_6_-PAA nanoparticles was not accompanied by an additional decrease in the number of cells, which adhered to the cell substrate regardless of the dose, in contrast to EMT6P carcinoma cells (Figure 4). We analyzed the data for the clonogenic assay and found a synergistic effect for 2 and 4 Gy at 25 and 50 μg/mL using the CDI criterion. For EMT6/P cells, the CDI for 25 and 50 μg/mL at 2 Gy was 0.16 and 0.48, respectively, and for BT474 cells, the CDI for 5 and 10 μg/mL was 0.86 and 0.62, respectively. Increasing the radiation dose to 4 Gy enhanced the synergistic effect. It should be noted that survival at 4 Gy was greater than at 2 Gy for 1 μg/mL, which has an inverse relationship with respect to the expected linear-quadratic model of the dose–response relationship (Figure 4b). Despite this trend, this result was not statistically confirmed. For EMT6/P cells, the CDI value for concentrations of 25 and 50 μg/mL at a dose of 4 Gy was 0.01 and 0.39, respectively, and for BT474 cells, at concentrations of 5 and 10 μg/mL, the CDI values were 0.36 and 0.45, respectively. Thus, it can be concluded that the combined action of lanthanum boride nanoparticles and a proton beam provides synergy in the implementation of their radiosensitizing effect.

**Figure 5:**
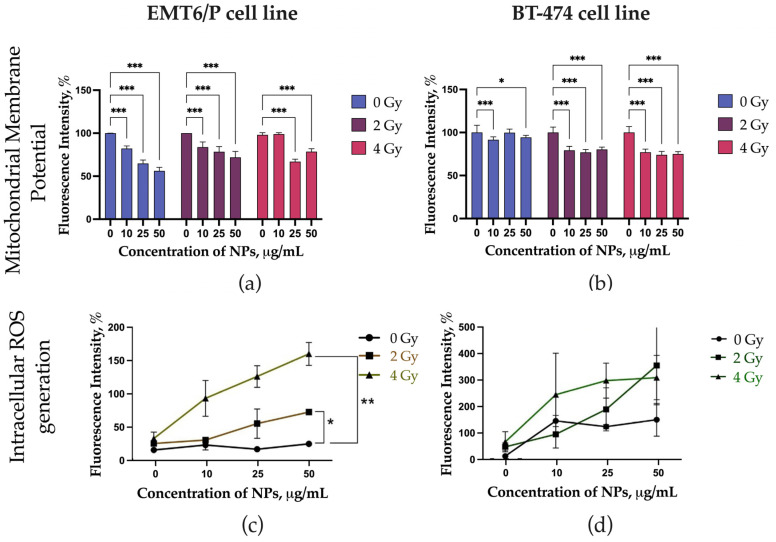
Analysis of the mechanisms of the radiosensitizing effect of LaB_6_-PAA NPs, including analysis of the level of mitochondrial membrane potential (MMP) on (**a**) EMT6/P cell line cultures and (**b**) BT-474 cell line cultures, and the level of intracellular ROS (Cell ROX staining) on (**c**) EMT6/P cell line cultures and (**d**) BT-474 cell line cultures at 72 h after irradiation with a proton beam at the Bragg peak. The statistical significance of deviations between the control and test samples is indicated by *, where * *p* < 0.05, ** *p* < 0.01 and *** *p* < 0.001.

The effects of LaB_6_-PAA nanoparticles combined with proton irradiation on the mitochondrial membrane potential and the level of intracellular ROS are shown at Figure 5. LaB_6_-PAA nanoparticles induced a decrease in MMP for EMT6/P cells even without irradiation, while BT-474 cells showed only proton-induced decrease in MMP at all studied concentrations. The concentration-dependent and dose-dependent decrease in MMP was observed for EMT6/P cells under the combined effect of proton irradiation and preincubation of the cells with LaB_6_-PAA nanoparticles. The concentration-dependent increase in the level of intracellular ROS in the presence of nanoparticles occurred after 72 h of coincubation. LaB_6_-PAA nanoparticles demonstrated the radiation-induced decrease in MMP for the BT-474 line, both the part of apoptotic cells and the level of intracellular ROS.

### 3.4. Safety

**Figure 6:**
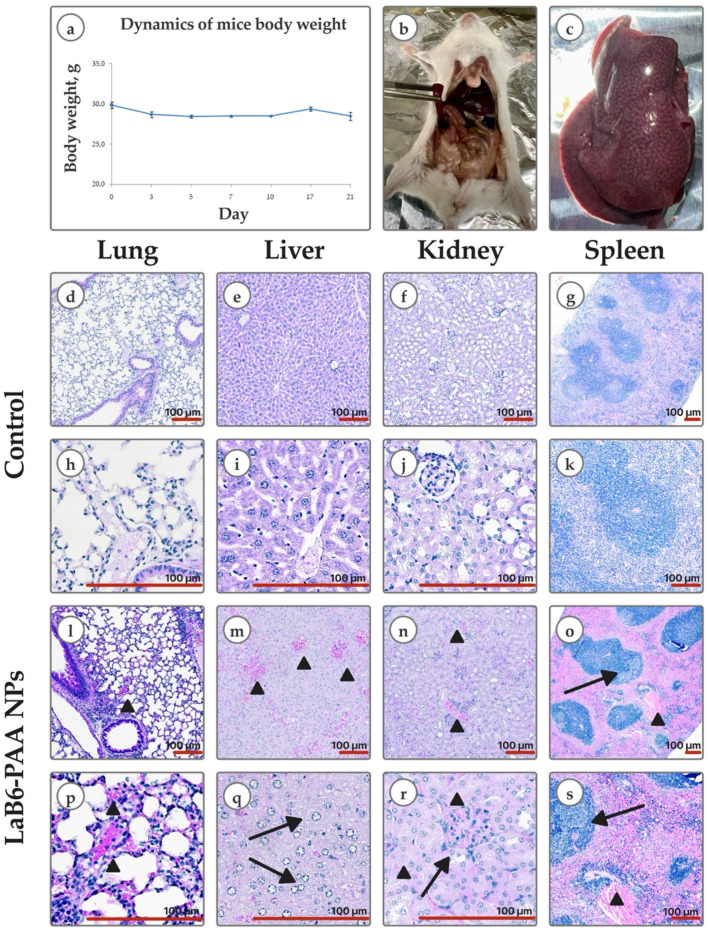
In vivo safety: (**a**) body weight dynamics of mice after a single intravenous administration of LaB6-PAA NPs at a dose of 0.1 mg/mouse. Graphical deviations correspond to SD. Gross pathology: (**b**) intestinal infarction; (**c**) nutmeg liver; (**d**–**k**) histological images (H&E) of lungs, liver, kidneys, and spleen of mice of the control group and mice that died after a single intravenous administration of LaB6-PAA nanoparticles at a dose of 2 mg/mouse (**l**–**s**). Lung: (**l**,**p**) full-blooded intra-alveolar arterioles and capillaries (arrowhead). Liver: (**m**) hepatocyte edema with narrowing of sinusoidal lumen, extra full-blooded central veins (arrowhead); (**q**) ballooning hepatocytes (arrow). Kidney: (**n**) full-blooded interstitial capillaries (arrowhead); (**r**) full-blooded glomerulus infiltrated with lymphocytes (arrow) and tubules with cell injury and necrosis (arrowhead). Spleen: (**o**,**s**) lymphoid follicle with small light germinative center (arrow), full-blooded red pulp (arrowhead). Scale bar—100 μm.

Taking into account the revealed in vitro signs of cytotoxic effect, we evaluated the safety of the systemic intravenous administration of LaB_6_-PAA NPs. I.v. administration of LaB_6_-PAA NPs to Balb/c mice at a dose of 0.1 mg/mouse did not noticeably affect the well-being and survival of mice for at least 21 days. A decrease in weight by up to 10% was observed only during the first 7 days of observation; subsequently, the dynamics of weight normalized (Figure 6). No gross changes in internal organs were found during necropsy on the 21st day of the study.

The intravenous administration of LaB_6_-PAA nanoparticles to Balb/c mice at a dose of 2 mg/mouse also did not cause an immediate negative effect. However, intoxication developed gradually. After several hours, gradually increasing depression of the animals, decreased skeletal muscle tone, and changes in reactions to external stimuli (from weakening to complete absence) were observed. The death of all animals occurred within 15–17 h after administration. Gross examination showed venous hyperemia of internal organs, “nutmeg liver”, and features of infarction of the upper intestine. Histological kidney specimen examination showed foci of tubular epithelial cell injury and cell necrosis, including classic coagulative necrosis and apoptosis, manifestations of sublethal injury and tibular exfoliation. Lung tissue contained full-blooded vessels; alveoli were infiltrated with lymphocytes. Histological findings in liver included lobular lymphocytic infiltrate, ballooning degeneration, and extremely full-blooded sinusoidal capillaries. Spleen red pulp was full-blooded; the white pulp was represented both by lymphoid nodules without light centers and with small light centers. The effects revealed are mainly manifestations of systemic hemodynamic disturbances of the microcirculature, which could be considered systemic signs of disturbance of calcium-dependent processes. The obtained results are consistent with the literature data describing similar systemic toxic effects for lanthanides, accompanied by vascular congestion and histopathological changes in the liver and gills in aquatic organisms [45] and in the spleen and kidneys of mammals [46,47], which allows us to associate them not so much with the NPs effect, but with the action of lanthanum, which is part of the nanoformulation.

### 3.5. Tolerability of Antitumor Therapy

**Figure 7:**
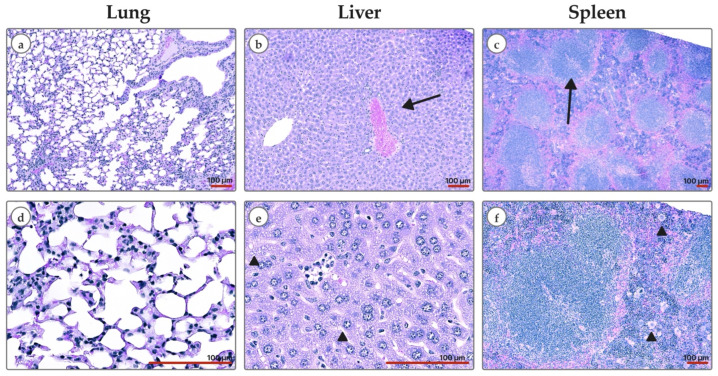
Histological images (H&E) of Nu/j mice on the 26th day after binary therapy with i.t. administration of LaB6-PAA NPs, followed by proton irradiation (31 Gy + 15 Gy): (**a**,**d**) lung tissue with preserved alveolar architecture and no signs of acute inflammation or fibrosis; (**b**) full-blooded central vein (arrow), hepatocyte congestion with narrowing of sinusoidal spaces; (**e**) enlarged and swollen ballooning hepatocytes with granular material in the cytoplasm (arrowhead); (**c**) the splenic architecture showed well-defined follicles with small generative centers (arrow); (**f**) presence of megakaryocytes in red pulp. Scale bar—100 μm.

I.t. injection of a colloidal solution of LaB_6_-PAA NPs at a dose of 2.0 mg/mouse to athymic Nu/j mice with a human tumor xenograft was well tolerated. No signs of intoxication or lethal outcomes were registered. A short-term, rapidly passing pain reaction in most mice was noticed at the injection site without further development of local toxic effects. Histologic specimen examination of animals on the 26th day after NP administration, followed by fractional proton irradiation, showed swelling of hepatocytes and vacuolar degeneration in their cytoplasm (Figure 7). The histoarchitecture of the spleen was characterized by the expansion of the white pulp and blurring of the boundaries of the white and red pulp due to cellular proliferation. The massive lymphocytic infiltration of the red pulp and the accumulation of megakaryocytes both along the periphery and in deeper parts of the organ were also observed. Histological features of lung tissue damage were not observed. Typical changes of the toxic effect of LaB_6_-PAA NPs were not noted.

### 3.6. Antitumor Efficacy In Vivo

**Figure 8:**
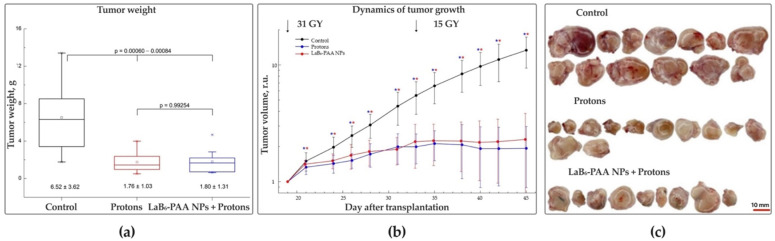
Comprehensive analysis of the efficacy of proton therapy (31 Gy + 15 Gy) in combination with i.t. administration of LaB_6_-PAA NPs: (**a**) Box diagrams of the distribution of the mass of BT-474 carcinoma xenografts in mice of the experimental groups on the 45th day of growth. (**b**) Analysis of the dynamics of neoplasia growth (indicators of neoplasia volume for each animal are normalized to the tumor volume at the start of radiation exposure (19th day); graphic deviations correspond to SD. The statistical significance of deviations between the control and treatment groups is indicated by *, where * *p* < 0.05. (**c**) Gross of tumor nodes. Scale bar: (**c**) 10 mm. One animal in the control group was prematurely removed from the study to prevent suffering.

The tumor model of a BT-474 carcinoma xenograft turned out to be very sensitive to proton irradiation. The necropsy of BT-474 carcinoma xenografts in mice of the experimental groups revealed small tumor nodes, visually expressed depletion of their peritumoral vascularization, and distinct macroscopic manifestations of dystrophic changes in tumor tissues (Figure 8). During the whole period of observation, the dynamics of the growth indices, as well as the data of terminal mass measurement of BT-474 carcinoma xenografts in the groups of mice that received binary therapy, did not statistically differ from the indices of the group that received only proton irradiation (Figure 8). An excessively high antitumor efficacy of proton radiation in both the proton exposure group and the group of animals receiving binary therapy was observed. That resulted in the severe alteration of experimental neoplasia tissues and the stable suppression of BT-747 xenograft growth with no statistically significant manifestations of recurrent growth during at least 26 days after the primary dose of irradiation. Excessive proton exposure did not allow for identifying significant morphometric manifestations of the LaB_6_-PAA NPs’ radiosensitizing effect for 26 days after exposure.

**Figure 9:**
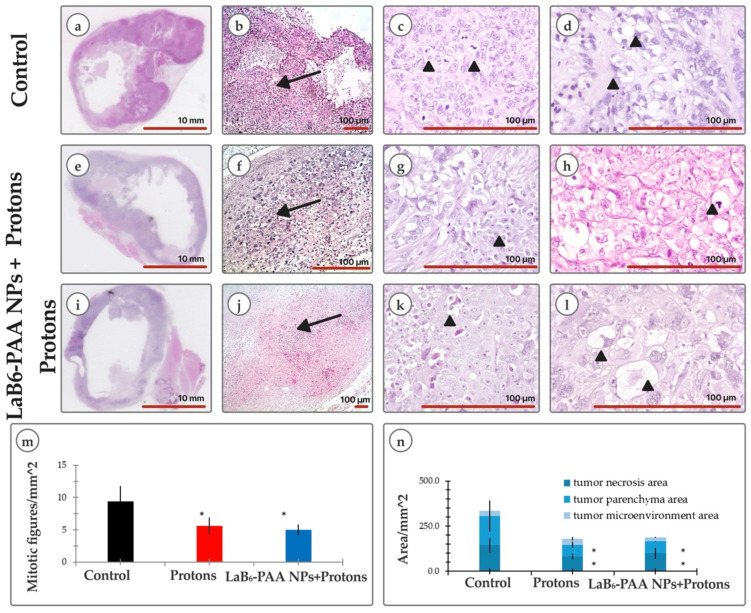
Morphology assay: (**a**,**e**,**i**) overview image of a cross section of a tumor node along the largest diameter (H&E); (**b**,**f**,**j**) parenchyma (arrow) of BT-474 tumor nodes in athymic Nu/j mice on the 45th day of growth of the corresponding groups (H&E); (**c**) mitosis of cancer cells (arrowhead); (**g**) nest of cancer cells with the presence of mitotic activity (arrowhead); (**k**) degenerative tumor cell with enlarged foamy cytoplasm (arrowhead); (**d**) tumor cells with optically empty cytoplasm; (**h**) apoptotic body among polymorphic tumor cells (arrowhead); (**l**) ballooning tumor cells with highly enlarged foamy cytoplasm; (**m**) analysis of mitotic activity; (**n**) morphological analysis of the tumor node microstructure, including morphometric analyses of tumor necrosis area, tumor parenchyma area, and tumor microenvironment area. The statistical significance of deviations between the control and treatment group, where * *p* < 0.05. Scale bar: (**a**,**e**,**i**) 10 mm; scale bar: (**b**–**d**,**f**–**h**,**j**–**l**) 100 μm.

The results of histological studies of BT-474 carcinoma xenografts also show the pronounced radiation alteration of the tumor in mice of the experimental groups and the development of similar deep dystrophic and necrotic changes induced by proton and binary effects. On the 45th day after transplantation, the BT-474 carcinoma xenograft in athymic Nu/j mice of the control group was a solid formation, the parenchyma of which was represented by typical moderately polymorphic epithelioid BT-474 cells with a homogeneous, in places, cleared cytoplasm and smooth rounded nuclei (Figure 9b–d,n). Dystrophic changes in the cells were minimal in a few areas. Foci of spontaneous necrosis accounted for about 30% of the tumor node area, which were determined mainly in the central part and were accompanied by signs of moderate inflammatory neutrophilic–eosinophilic infiltration. In the tumor nodes of the BT-474 carcinoma xenograft in mice that received only proton irradiation without the use of combined therapy approaches, a pathomorphological picture of pronounced radiation alteration of neoplasia was observed. Necrotic zones occupied up to 50% of the section area. For the most part, the parenchyma was represented by the fields of dystrophically altered, atypical, sharply polymorphic cells. Morphotypic BT-474 cells in the tumor parenchyma were detected as small foci (Figure 9f–h,n). In histological sections of tumor nodes of the BT-474 carcinoma xenograft in mice that received binary therapy with LaB_6_-PAA nanoparticles followed by proton irradiation, the pathomorphological picture of a pronounced radiation alteration of neoplasia was observed as well, but with more extensive (up to 60% of the tumor node area) foci of induced necrosis (Figure 9j–l,n). The parenchyma was presented by 2–3 populations of atypical polymorphic cells with pronounced dystrophic changes. Typical BT-474 cells were visualized as single foci, and were not visualized on individual preparations. That can be considered a sign of a more pronounced slowdown in the recurrent growth of BT-474 carcinoma after proton irradiation in the presence of LaB_6_-PAA NPs, compared with proton irradiation, in which case foci of morphotypic epithelioid cells were recorded more often. Histological signs of increased tumor cell migration beyond the tumor node were not noted. The proliferative activity of tumor cells in both groups was significantly reduced as compared with the control group (Figure 9m). At the same time, the mitoses, as a rule, were atypical, which was the evidence of reproductive cell death.

### 3.7. Biodistribution

**Figure 10:**
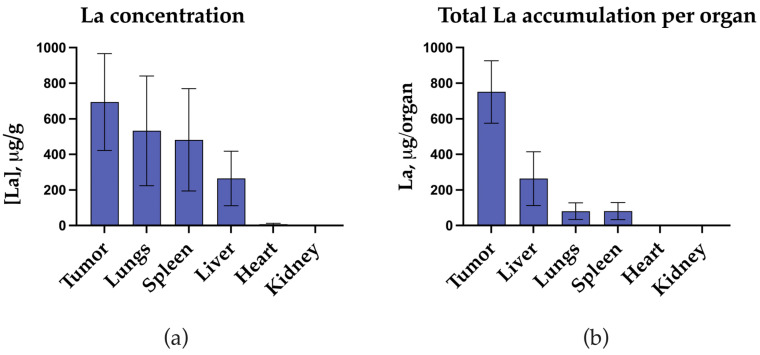
Bioavailability of NPs by day 26 after a single i.t. administration: (**a**) average accumulation of lanthanum per mouse organ; (**b**) concentration of lanthanum in mouse organs. Data are presented as mean ± SD, *n* = 5.

Systemic bioavailability from the injection site to the organs and tissues of the tumor-bearing animal, measured on the 26th day after injection, averaged 21% of the administered dose. These data demonstrate partial redistribution of lanthanum from the tumor after intratumoral administration of nanoparticles. The distribution we observed was typical for nanoparticles, with high accumulation in organs rich in phagocytic cells: liver, lungs, and spleen (Figure 10).

## 4. Discussion

A widely developed approach to increasing the efficacy and safety of radiotherapy lies in the use of selectively accumulated nanosized sensitizing formulations and drugs based on them in the tumor. The search for sensitizer materials to increase the relative biological effectiveness of proton irradiation includes a comprehensive study of the candidate material. It results in the analysis of the ratio of the efficacy and safety of the material. The obtained LaB_6_-PAA nanoparticles demonstrated the promising size and hydrodynamic characteristics, which, in combination with a low work function (2.50 eV), and degradability in aqueous conditions, as well as a relatively high atomic number of lanthanum (Z = 57), allows us to consider them to be a radiosensitizing agent for increasing the biological effectiveness of proton therapy. When evaluating the effectiveness of proton irradiation on BT-474 cell lines after co-incubation with LaB_6_-PAA nanoparticles, the increase in the presence of radiation-induced cytotoxic effects of proton therapy was shown: the decrease in MMP values, as well as the increase in the number of dead and apoptotic cells and the level of intracellular ROS, which also confirms the sensitizing potential of the formulation.

The important aspect of our in vivo experimental design is the use of a spread-out Bragg peak (SOBP) proton field, as opposed to a single pristine Bragg peak. Clinically, an SOBP is created by superposing proton beams of different energies to achieve a uniform dose over the tumor volume. In our case, a 1.5 cm SOBP (centered at ~100 MeV) was used, yielding a fairly homogeneous dose region with proton LET spanning approximately 7 to 14 keV/μm. This has practical significance. It suggests that, within a clinical SOBP field, nanoparticles can bolster the therapeutic effect throughout the target volume, without requiring preferential deposition at the Bragg peak maximum.

Our results are in line with other investigations that have moved beyond idealized beam setups to more clinical ones. For example, recent cell studies with gold nanoparticles have also employed SOBP irradiation and reported significant sensitization, comparable to or greater than that seen with single-energy beams [48]. SOBP delivery inherently involves a trade-off: the peak LET at the distal edge is lower than that of a pristine Bragg peak (since the pristine peak is spread out), but in return, the dose is distributed to cover the entire tumor depth uniformly.

Additionally, multiple studies indicate that nanoparticle radiosensitization efficiency can depend strongly on the linear energy transfer (LET) of the radiation. High-Z nanoparticles like gold have shown minimal enhancement under low-LET proton irradiation but significant effects at higher LET. For example, Li et al. observed a marked radiosensitization with 25 keV/μm protons, whereas 10 keV/μm protons yielded no significant nanoparticle effect [28]. This suggests a threshold or optimal LET range above which nanoparticle-mediated enhancement becomes pronounced. Our proton SOBP field (LET ≈ 7–14 keV/μm) lies between those values, aligning with an LET regime where moderate yet appreciable radiosensitization is expected. For context, this LET range is an order of magnitude higher than that of entrance (plateau) high-energy protons (~1 keV/μm for 150 MeV protons [49]) and is on par with or slightly above the LET of conventional therapeutic proton fields in mid-depth tissues. Thus, using an SOBP likely positioned the nanoparticles in an intermediate LET environment conducive to enhancement, without the need for extreme distal-edge positioning.

The results of the evaluation of the effectiveness of the radiosensitizing effect of LaB_6_-PAA NPs on the BT-474 carcinoma xenograft model in Nu/j athymic mice in the experiment were less pronounced due to the high sensitivity of the BT-474 carcinoma xenograft to proton irradiation in the study, which resulted in tissue hyperalteration.

Proton therapy is an emerging and rapidly developing modality in the field of radiotherapy. This results in a shortage of data on the sensitivity of different tumor cell lines to proton irradiation. The limited studies available demonstrate significant variability in treatment outcomes, which appear to depend strongly on the irradiation mode and parameters. For example, in [50], the authors explored the effects of radiotherapy on the subsequent growth of tumor derived from various lines, using doses ranging from 5 Gy (fractional exposure 3 Gy + 2 Gy) to 50.6 Gy. The findings revealed considerable variation in tumor response, emphasizing the need for more comprehensive and systematic studies on cell line-specific sensitivity to proton therapy. In [51], a triple sequential irradiation of the BT-474 xenografts in Balb/c mice at a dose of 3 Gy (a total of 9 Gy) using a 6 MeV electron beam with a 10 mm diameter was performed. The treatment did not result in any significant tumor growth inhibition compared with the control group. Tumor progression in the groups subjected to the irradiation was similar to that in the control group, suggesting that the radiation dose applied in a study by Park Y. was insufficient to achieve a measurable therapeutic effect. However, it should be emphasized that the presented data represent the first radiobiological study evaluating the efficacy of proton irradiation in vivo using the BT-474 xenograft model in athymic mice. The proton irradiation doses were selected based on our previous studies [21] involving an allograft model of solid Ehrlich carcinoma, in which only a modest radiation-induced alteration of neoplasia was observed under a comparable irradiation regime (protons, 31 Gy). This allowed the additional biological effectiveness of potential sensitizers to be more clearly detected, without being masked by a dominant radiation effect. The results of morphometry obtained in the present study showed a higher sensitivity of the BT-474 carcinoma xenograft to proton irradiation. Instead of the expected transient inhibition of tumor growth, which is followed by the subsequent restoration of the proliferative potential and the recurrent growth of tumor nodes at the rate typical of the intact tumor, we observed a more important tissue alteration. Within 2 days following the onset of exponential growth, the first proton irradiation induced a sharp and sustained suppression of BT-474 xenograft growth, with the absence of statistically significant manifestations of recurrent growth. The regression of the BT-474 xenograft caused only by proton irradiation at a dose of 31 Gy nearly corresponded to a complete inactivation of neoplasia with the loss of its ability to respond to more intense antitumor effects in this experiment. This conclusion is supported by the lack of significant changes in the dynamics of morphometric parameters of the carcinoma nodes after repeated irradiation at a dose of 15 Gy, 14 days after the first irradiation. The revealed features of radiosensitivity of the VT-474 xenograft to a more severe proton irradiation regimen on the model used inevitably limited the ability to detect morphometric signs of the radiosensitizing effect of the studied nanoparticles.

The obtained result is undeniably important, as it confirms the individual sensitivity of tumor lines to proton irradiation, underscores the sensitivity of the method for evaluating the effectiveness of antitumor radiotherapy, and highlights the critical need for precise titration of radiation doses for each tumor type, both to minimize limitations in experimental design and to optimize therapeutic outcomes in clinical practice.

Although direct morphometric evidence of enhanced antitumor effects of binary therapy was not observed, morphological analysis revealed signs of reduced proliferative activity compared with proton irradiation alone. In light of these promising radiosensitizing effects, it is important to note that lanthanum hexaboride nanoparticles functionalized with polyacrylic acid (LaB6-PAA) also exhibit notable cytotoxicity. In vitro preincubation of LaB6-PAA nanoparticles with EMT6/P and BT-474 cells, in the absence of irradiation, led to a reduction in cell viability—partly attributed to diminished adhesive properties. Here, BT-474 cells exhibited the lowest sensitivity to the cytotoxic effects of LaB_6_-PAA nanoparticles. Given the similarity of the identified effects with the action of chelating agents on adhesive cell cultures, it can be hypothesized that the identified effects involve a cadherin-mediated mechanism. Considering the known degradation of LaB_6_ nanoparticles in aqueous environments, the cytotoxic effects are likely linked to the release of lanthanum ions. Owing to their close ionic radii and similar coordination chemistry, lanthanum ions exhibit a strong chemical affinity for calcium-binding sites and can substitute calcium in biological systems. This substitution enables lanthanum to competitively bind to Ca^2^⁺-dependent receptors with high affinity. As a result, several calcium-dependent cellular processes may be disrupted—some may be inhibited by the displacement of Ca^2^⁺, while others may be aberrantly activated—thereby contributing to the cytotoxic profile observed [52]. To achieve a functional conformation, cadherins require Ca^2+^ binding at all binding sites. The loss of Ca^2+^ binding or, in the case of exposure to lanthanum ions, replacement leads to a violation of the protein conformation and a violation of cadherin-mediated cell adhesion. In other words, chemically replacing Ca^2+^ in cadherins, lanthanum cannot replicate it functionally. As a result, cadherins associated with it lose their ability to maintain cellular adhesion [53,54]. A similar effect was demonstrated earlier in vitro by L. Brayshaw et al. for terbium ions Tb^3+^ from the lanthanide group [55]. Furthermore, Nikolova V. et al. systematized the ability of the entire series of lanthanides to replace Ca^2+^ in biological systems [56].

Given the properties of the studied NPs, it is important to consider the role of cadherins in the mechanisms of cell adhesion of tumor tissues. This role largely depends on the cellular context and is associated with the process of metastasis, as shown in several cancer models [38,39]. Both the loss and overexpression of cadherins can critically influence the acquisition of invasive and metastatic potential by cancer cells. For instance, E-cadherin maintains cell adhesion in the tumor, while its loss increases invasion in various models of invasive ductal carcinomas. However, the loss of E-cadherin may also lead to reduced cancer cell proliferation, survival, and diminished efficiency in metastatic seeding and outgrowth [57]. In melanoma, non-small cell lung carcinoma, squamous cell carcinoma of the oral cavity, and hepatocarcinoma, P-cadherin has a tumor suppressor behavior similar to E-cadherin. While an aberrant expression of P-cadherin associated with aggressive tumor behavior is observed in breast, gastric, endometrial, ovarian, prostate, pancreatic, bladder, and colorectal carcinomas [58], a common feature of the metastasis process is the loss of epithelial cadherin (E-cadherin). This suppresses tumor cell migration and the expression or overexpression of neural cadherin (N-cadherin), which stimulates tumor cell invasiveness [59]. In our study, we found that the co-cultivation of EMT6/P and BT-474 cells with the studied nanoparticles leads to the disruption of cell adhesion, which in vivo may be accompanied by an increase in the metastatic potential of neoplasia. However, in the study of the effectiveness of combination therapy on the BT-474 carcinoma xenograft model in athymic Nu/j mice after i.t. administration of LaB_6_-PAA NPs, we found no confirmation of this. In addition to the cytotoxic effect, a pronounced acute toxic effect of LaB_6_-PAA NPs was observed following systemic intravenous administration of colloidal solutions to mice at a dose of 2.0 mg/mouse. This led to the death of animals (LD_100_) during the first day, as a result of a complex vascular reaction, compounded by the blockade of calcium-dependent processes. At the same time, intratumoral administration of LaB_6_-PAA NPs at a similar dose of 2.0 mg/mouse was not accompanied by the development of similar signs of intoxication of the body, which is associated with the predominant deposition of NPs at the site of administration and a low level of systemic availability.

Gross and histological examinations of dead animals, along with data on systemic bioavailability from the injection site and the results of evaluating the nanoparticle degradation under biorelevant conditions, suggest that the observed toxic effects are primarily attributed to the action of lanthanum, a component of the nanoparticle formulation, rather than the nanoparticles themselves. Due to the high level of bioavailability upon intravenous administration, a large proportion of particles become available for the internalization by the body’s cells (including phagocytic cells), where active degradation of NPs occurs, with the release of lanthanum ions. Subsequently, lanthanum enters the systemic bloodstream, triggering the development of intoxication. Taking this into account, the further direction of development of nanoformulations based on lanthanum hexaboride should be focused on reducing the systemic toxicity of these particles, potentially through the addition of a protective coating that slows their degradation under biorelevant conditions.

## 5. Conclusions

Thus, we investigated the efficacy of using colloidal solutions of laser-synthesized lanthanum hexaboride (LaB_6_) nanoparticles (NPs) coated with polyacrylic acid (PAA) as sensitizers to increase the anti-tumor biological effectiveness of proton irradiation. In vitro data demonstrated the efficacy LaB_6_-PAA NPs at a concentration above 1 μg/mL. In the in vivo model of a BT-474 carcinoma xenograft in athymic Nu/j mice, with i.t. administration at a dose of 2.0 mg/mouse, the effect was less pronounced due to the high sensitivity of the xenograft to proton irradiation, and further studies are needed to validate the therapeutic applicability. Moreover, despite the identified radiosensitizing effect, LaB_6_-PAA NPs exhibited significant cytotoxicity in vitro at concentrations above 5 μg/mL, as well as considerable systemic toxicity in vivo at i.v. administered at a dose of 2.0 mg/mouse. This demonstrates a narrow therapeutic index, significantly limiting the prospects for using these particles. A possible way to reduce their toxicity may be additional surface modification to prevent rapid degradation of NPs under biorelevant conditions. The effectiveness of this approach, however, still requires further validation.

## Data Availability

All the data are presented within this article. The raw datasets are available on request from the corresponding authors.
